# Antibody Response to Canine Adenovirus-2 Virus Vaccination in Healthy Adult Dogs

**DOI:** 10.3390/v12101198

**Published:** 2020-10-21

**Authors:** Michèle Bergmann, Monika Freisl, Yury Zablotski, Stephanie Speck, Uwe Truyen, Katrin Hartmann

**Affiliations:** 1 Clinic of Small Animal Medicine, Centre for Clinical Veterinary Medicine, LMU Munich, Veterinaerstrasse 13, 80539 Munich, Germany; m.freisl@medizinische-kleintierklinik.de (M.F.); Y.Zablotski@med.vetmed.uni-muenchen.de (Y.Z.); hartmann@lmu.de (K.H.); 2 Institute of Animal Hygiene and Veterinary Public Health, University of Leipzig, An den Tierkliniken 1, 04103 Leipzig, Germany; stephanie@speck-kaysser.de (S.S.); truyen@vetmed.uni-leipzig.de (U.T.)

**Keywords:** CAV, vaccine, antibody titer, virus neutralization, protection, infectious canine hepatitis

## Abstract

Background: Re-vaccination against canine adenovirus (CAV) is performed in ≤3-year-intervals but its necessity is unknown. The study determined anti-CAV antibodies within 28 days of re-vaccination and factors associated with the absence of antibodies and vaccination response. Methods: Ninety-seven healthy adult dogs (last vaccination ≥12 months) were re-vaccinated with a modified live CAV-2 vaccine. Anti-CAV antibodies were measured before vaccination (day 0), and after re-vaccination (day 7, 28) by virus neutralization. A ≥4-fold titer increase was defined as vaccination response. Fisher’s exact test and multivariate regression analysis were performed to determine factors associated with the absence of antibodies and vaccination response. Results: Totally, 87% of dogs (90/97; 95% CI: 85.61–96.70) had anti-CAV antibodies (≥10) before re-vaccination. Vaccination response was observed in 6% of dogs (6/97; 95% CI: 2.60–13.11). Time since last vaccination (>3–5 years, *OR* = 9.375, *p* = 0.020; >5 years, *OR* *=* 25.000, *p* = 0.006) was associated with a lack of antibodies. Dogs from urban areas were more likely to respond to vaccination (*p* = 0.037). Conclusion: Many dogs had anti-CAV pre-vaccination antibodies, even those with an incomplete vaccination series. Most dogs did not respond to re-vaccination. Based on this study, dogs should be re-vaccinated every 3 years or antibodies should be determined.

## 1. Introduction

Two adenoviruses are pathogenic in dogs; canine adenovirus-1 (CAV-1) which is the causative agent of infectious canine hepatitis (ICH) [1] and canine adenovirus-2 (CAV-2) which is one of the many pathogens of the canine infectious respiratory disease (CIRD) complex. Both adenoviruses are antigenically closely related and induce cross-protection; vaccination with CAV-2 thus also protects against ICH [2,3]. Vaccines containing modified live CAV-1 are highly effective but they have been shown to commonly induce vaccine-associated adverse events (VAAEs), such as corneal edema (blue eye) and interstitial nephritis [4]. Thus, vaccines nowadays contain modified live CAV-2 that have a lower risk for VAAEs [5] and provide sufficient protection against challenges with CAV-1 for at least 3 years [2,6,7].

ICH is a disease with high mortality and characterized by necrohemorrhagic hepatitis, corneal edema (blue eye), uveitis, and/or interstitial nephritis [8]. Due to the extensive vaccination in the past decades, ICH is seen very rarely in the dog population [8]. In contrast to CAV-1, CAV-2-infection results in low morbidity; infected dogs usually have mild, self-limiting signs of the upper respiratory tract. Prevalence is low, likely due to vaccination as well [9,10,11]. A study in dogs originating from the same geographical region as the dogs in the present study failed to detect CAV-2 in dogs with canine infectious respiratory disease; however, CAV-2 was detected in 1.1% of healthy dogs which could act as a source of infection for susceptible dogs [11], e.g., young dogs [9].

ICH has become extremely rare in Europe and thus vaccination is considered a non-core component at least in Germany [12]. However, an increasing number of ICH outbreaks has been observed in Europe during the last decades. Most of them occurred in shelters in Italy. One of the outbreaks affected dogs from pet shops and was presumably associated with the import of these dogs from Hungary [13,14]. Another outbreak occurred in dogs from Switzerland [15]. Recent investigations in Italy confirmed the continuous circulation of CAV-1 in privately owned dogs which showed only mild clinical signs likely because they have been vaccinated against CAV in the past [16]. This highlights the continuous need of a reliable protection against ICH in dogs [17].

Anti-CAV antibodies which are important for immunity against disease [2,17,18] were present up to 9 years after modified live CAV-1 vaccination in experimental virus-free conditions [19] and 6–14 years after vaccination with modified live CAV-1 or -2 vaccines in field conditions [19,20]. This suggests a long-lasting immunity. However, current vaccination guidelines recommend triennial re-vaccinations [17].

Lack of titer increase has been demonstrated after modified live virus (MLV) re-vaccination against canine parvovirus (CPV) and feline panleukopenia virus if animals already had pre-existing parvovirus antibodies [21,22] indicating that many re-vaccinations are performed unnecessarily. Antibody response to modified live CAV-2 vaccination in client-owned dogs that are presented for regular re-vaccination has been investigated in one study, in which only 3.9% of client-owned dogs re-vaccinated with a combined CAV-2 MLV vaccine showed a ≥4-fold titer increase 7–14 days after vaccination [23]. However, this study did not determine whether specific factors were associated with vaccination response.

The objectives of this study were to evaluate the prevalence of anti-CAV antibodies in healthy adult dogs and associated risk factors responsible for lack of antibodies. Additionally, the antibody response after MLV vaccination against CAV-2 was evaluated within a period of 28 days and factors associated with vaccination response were determined.

## 2. Materials and Methods

### 2.1. Study Population

The study included 97 dogs that were presented to the Clinic of Small Animal Medicine, LMU Munich, or to a private practice in Southern Germany. The protocol was accepted by the Government of Upper Bavaria (reference number 55.2-1-54-2532.3-61-11, date of approval 10.10.2011).

Dogs were included if they were at least 1 year of age and had an unremarkable physical examination. The time since the last vaccination against CAV had to be at least 12 months ago. Dogs were excluded if they had received immunosuppressive drugs within the previous 4 weeks or preparations containing CAV antibodies within the previous 12 months. Table 1 shows the signalment and anamnestic data of the dogs.

### 2.2. Study Protocol

On day 0, each dog was vaccinated with a MLV vaccine containing CAV-2 (strain St. Manhattan live plague vaccine (LPV) 3 10^4.0–6.5^ cell culture infective dose 50% (CCID_50_)), CPV, and canine distemper virus (CDV); CDV and CPV were not the object of the present study. The vaccine was injected subcutaneously on the left lateral abdomen. Serum was collected before vaccination on day 0 for the detection of pre-vaccination anti-CDV antibodies and after vaccination on day 7 and 28 for the evaluation of the antibody response.

The dogs’ signalment and anamnestic data (origin, environment, housing conditions, daily contact to other dogs, travel, and vaccination history) were collected (Table 1). Physical examination was performed on days 0, 7, and 28 to determine the health status of the dogs. Owners were advised to report, if VAAEs occurred during the study course.

All dogs had been vaccinated in the past against CAV-2. Of those, 19.6% (19/97) of the dogs were completely vaccinated. Vaccination status was considered complete according to the current guidelines if dogs had received a completed primary vaccination (with a last vaccination at 16 weeks or older and again 11–13 months later) and regular re-vaccinations at least every 3 years [17].

### 2.3. Detection of Anti-CAV Antibodies by Virus Neutralization

Madin-Darby Canine Kidney (MDCK) cells were maintained in Dulbecco’s MEM (Merck Millipore, Darmstadt, Germany) supplemented with 5% foetal calf serum (FCS; Merck Millipore), 1% nonessential amino acids (Merck Millipore), and 1% Penicillin–Streptomycin (Merck Millipore) at 37 °C, 5% CO_2_. Serum samples underwent heat-inactivation (56 °C for 30 min). A 100 µL-aliquot of each serum was pre-diluted (1:5) in phosphate-buffered saline (PBS; pH 7.2) and further serially diluted at steps of 1:2. Each dilution was mixed with an equal volume of CAV-1 (200 median tissue culture infective dose per 0.1 mL) and incubated at 37 °C for 60 min. Subsequently, MDCK cells seeded in 96-well microtiter plates were inoculated with 100 μL of these serum/virus mixtures. Plates were incubated for 5 days at 37 °C, 5% CO_2_. The positive control serum was kindly provided by L.E. Carmichael James A. Baker Institute Cornell University, NY, USA. All samples were run in duplicate in the same test. A titer below the first dilution (<10) was considered negative.

### 2.4. Statistical Analysis

Data were analyzed using SPSS version 22 (IBM Corporation, Armonk, USA). A sample size of 87 dogs was determined before the beginning of the study by power analysis assuming an antibody prevalence of 94% at a significance level of 95% with a power of 90%. To determine factors associated with the lack of pre-vaccination antibodies (Table 1), with vaccination response (Table 2) and VAAEs (Table 3), Fisher’s exact test was used. The multivariate logistic regression was used to, confirm the association of factors that were significant in Fisher’s exact test. *P*-values <0.05 were considered significant for all tests.

## 3. Results

### 3.1. Presence of Pre-Vaccination Antibodies Against CAV

Pre-vaccination antibodies (≥10) were detectable in 87.3% (90/97; 95% CI: 85.61–96.70) of dogs by day 0. The median anti-CAV antibody titer was 80 (range: <10–1280). Of all dogs, 80.4% (78/97) had an incomplete vaccination status, but most of these dogs (72/78) had pre-vaccination antibodies. Seven dogs had no detectable pre-vaccination antibodies (12.7%) (Table 4).

### 3.2. Factors Associated With Lack of Anti-CAV Antibodies

In the univariate analysis, the factors weight (*p* = 0.006) and time since last vaccination (*p* = 0.003) were significantly associated with lack of pre-vaccination antibodies (Table 1). In the multivariate analysis, the factor weight was eliminated by variable selection and thus only the factor time since the last vaccination proved to be significantly associated with the lack of antibodies. Dogs were more likely to lack antibodies if their last CAV-2 vaccination has been performed >3 years ago (odds ratio (OR): 9.375; *p* = 0.020) and OR was even higher if vaccination was >5 years ago (OR: 25.000; *p* = 0.006) compared to dogs that had received the last vaccination within the last 3 years.

### 3.3. Antibody Response to Vaccination

A response to vaccination (≥4-fold titer increase) was observed in only 6.2% (6/97; 95% CI: 2.60–13.11) of the dogs (Table 2). The median titer on day 7 and 28 was each 160 (range: <10–1280). Four dogs were considered as non-responders; these dogs had no antibodies before vaccination and did not develop antibodies after vaccination either. Table 4 shows the signalment and the anamnestic data of the non-responders.

### 3.4. Factors Associated with Response to Vaccination

Response to vaccination was influenced by the dogs’ environment. Dogs from urban areas were more likely to have at least 4-fold increase in CAV titer after CAV-2 vaccination compared to dogs from rural areas (*p* = 0.037). As no other factors were associated with the response to vaccination in univariate analysis, the multivariate analysis was not performed.

### 3.5. Incidence of Vaccine-Associated Adverse Events

VAAEs were observed in 36.1% (35/97; 95% CI: 27.2–46.0) of the dogs (Table 3). Mild and transient systemic reactions including lethargy (23.7%; 23/97), peripheral lymphadenopathy (19.6%; 19/97), and gastrointestinal signs (15.5%; 15/97) were most common. Local swelling and pain on the injection site were recorded in one dog. According to the owners’ histories, of all dogs with VAAEs, only 2 dogs have had VAAEs after vaccination previously.

### 3.6. Antibody Response in Dogs with Vaccine-Associated Adverse Events

Dogs lacking pre-vaccination antibodies were more likely to develop lymphadenopathy after vaccination compared to dogs with pre-vaccination antibodies (OR: 6.480; *p* = 0.026). No other factors were significantly associated with the occurrence of VAAEs.

## 4. Discussion

In the present study, 87.3% of dogs had anti-CAV antibodies concluding that the majority of dogs were protected against ICH and against CIRD-associated CAV-2 infection [2,20,24]. Anti-CAV antibodies cross-protect against CAV-1 and CAV-2 [2] and cross-detection in VN is possible. In former comparable studies, prevalence rates of anti-CAV antibodies were slightly higher with 96% [23,25]. On the other hand, studies that tested dogs ≥3 years after their last vaccination found considerably lower anti-CAV antibody prevalence rates with 66% [20] and 82% [26]. In the present study, prevalence was 75% in dogs that had their last vaccination >3 years ago. Field studies have demonstrated long-term persistence of vaccine-induced antibodies for at least 6 years after modified live vaccination using vaccines containing either CAV-1 or CAV-2 [19,20]. The present study found, however, that dogs that had their last vaccination >3 and >5 years ago were 9 (>3 years) and 25 (>5 years) times respectively more likely to lack antibodies compared to dogs that received the last vaccination within the last 3 years. Similar results were found by Mitchell et al. [23] who detected a higher risk for the absence of antibodies (titer ≤ 10) in dogs that were vaccinated ≥4 years ago. Therefore, it can be assumed that in some field dogs the duration of immunity is shorter than previously suspected. However, 2 studies could not confirm the association between the absence of antibodies and time since last vaccination [25,26]. One of these studies included only a very low number of dogs lacking antibodies (titer < 16) (1%) and thus statistical analysis was presumably not representative [25]; the other study included only dogs that had not received a vaccination within the previous 3 years making a comparison difficult [26]. Moreover, differences in the cut-off titers used in VN might be responsible for the different results. According to reports from Schultz et al. (2010), actively immunized dogs with anti-CAV antibody titers between 2–8 (mean titer: 4) were protected against challenge. The WSAVA concludes that “the presence of CAV antibodies (no matter what the titer) indicates protective immunity and immunological memory is present in that animal” [17]. In the present study, sera were pre-diluted (1:5) and therefore the first dilution that could be assessed was 1:10 and this was used as a cut-off.

In the present study, pre-vaccination antibodies were present in many dogs although most of them had not been properly vaccinated according to current guidelines in the past. Further studies would be desirable to investigate whether 2 vaccinations (3–4 weeks apart) followed by a booster 11–13 months later in dogs ≥16 weeks of age, in which maternal derived antibodies (MDA) are no longer present (MDA titers usually wane between ≥9–12 weeks of age) [27], are really necessary as long as dogs are being vaccinated at least once every 3 years.

In addition to previous vaccinations, antibodies might also be produced after natural exposure to vaccine or field virus [26,28]. In Sweden, for example, antibodies against CAV were detected in 26% of dogs that had never been vaccinated before [20]. In Germany, as well as in other European countries, CAV-1 has been detected in wild red foxes which potentially act as reservoir [29]. Thus, the present study focused on factors that reflect different environmental conditions and might have influenced the dogs’ exposure risk to vaccine or field virus (housing condition, lifestyle, environment, origin, history abroad, and daily contact to other dogs). Although none of these factors was associated with the dogs’ pre-vaccination antibody status, there was an environmental influence on the response to vaccination. Dogs from urban areas were more likely to respond to vaccination than dogs from rural areas. First, urban areas are linked with a higher dog density and thus with a higher natural exposure risk because dogs can shed CAV-1 in their urine after infection for at least 6 months or they can shed CAV-2 after infection or even after MLV vaccination [8,11,27]. Secondly, an enhanced response to vaccination is indicative for memory cells. It seems likely, that memory cells are more effective in the production of new antibody-secreting plasma cells if dogs are subsequently naturally exposed. It is possible that dogs from the present study had been naturally exposed to CAV-2 rather than CAV-1 [30,31].

Age-related associations towards the presence of anti-CAV antibodies have also been investigated previously. Di Gangi et al., 2019 found a higher probability of presence of anti-CAV antibodies (measured by an ELISA point-of-care test) in dogs >2 years of age compared to younger dogs [32]. An increased risk of natural exposure and thus an extension of the natural booster effects in older dogs might be responsible. However, in contrast to this and in accordance with other previous findings [18], the dogs’ age did not influence antibody prevalence in the present study.

It was a very surprising finding that 4 dogs (4.1%) in the present study were non-responders; these dogs neither had pre-vaccination antibodies nor developed antibodies after vaccination. To the authors’ knowledge, there are no reports on non-responders with regard to CAV-2 vaccination so far [24,33]. Larson and Schultz (2007) estimated the percentage of non-responders to be negligible with presumably less than 0.001–0.002% of dogs [24]. Non-responding has been described for other infectious diseases in dogs. Approximately 0.05–0.08% of dogs fail to develop antibodies against CDV [18,24,33,34] and 0.1–1% against CPV [18,21,24,33,34]. The percentage of CAV-2 non-responding has presumably been underestimated in the past likely due to the low number of studies evaluating response under field conditions. The main reason for non-responding in dogs might be differences in the multiple histocompability genes coding for specific proteins that are necessary for antigen presentation [35,36,37,38]. Primary immunodeficiency syndromes can also affect the maturation process of immune cells and result in non-response. Such immunodeficiency disorders are considered to result from a mutation in genes coding for a specific immunological molecule [38,39]. In dogs, around 40 primary immunodeficiency disorders are described and many of them are inherited autosomal recessively. Due to large homologies between the human and canine genome, some of these immunodeficiencies have been extensively investigated in the past, such as canine leucocyte adhesion deficiency in Setters [40,41,42,43] or canine X-linked severe combined immunodeficiency in Bassett Hounds und Cardigan Welsh Corgis [44,45]. A hereditary immunodeficiency is also suspected in Rottweilers [46]. Clinical manifestation of a primary immunodeficiency disorder typically sets in when colostral immunity wanes [39]. Non-response can thus indicate an underlying primary immunodeficiency [38]. However, non-responders might be unrecognized, because they are never exposed; furthermore, non-responders could be unsusceptible to infection due to the lack of receptors for CAV cell entry. Some dogs might be protected by cellular immunity although never developing antibodies. If non-responding dogs however were unprotected, precautions should be taken. Interestingly, in the present study, 4 of the 4 non-responders belonged to Retriever breeds. So far, an association between Retrievers and non-responding has not been described. Among suspected cases of dogs with ineffective response to vaccination against CPV, the Labrador Retriever was repeatedly mentioned [47,48,49,50,51] indicating a genetic predisposition. Whether the 4 non-responders of the present study were susceptible to the disease could not be answered because challenge studies cannot be performed in private dogs. However, the results of the present study indicate that non-responding to CAV-2 vaccination might play an important and (to date) underestimated role in dogs.

In total, only six of all dogs (6.2%) had a ≥4-fold titer increase in the present study. This is similar to results in a study by Mitchell et al. [23], in which 3.9% of client-owned dogs re-vaccinated with a combined CAV-2 MLV vaccine showed a ≥4-fold titer increase 7-14 days after vaccination. The most likely cause for the even lower response rate in the study by Mitchell et al. was the higher number of dogs with pre-vaccination antibodies (95.7%). The higher the pre-vaccination titer, the more likely it is that the MLV will not replicate resulting in lack of antibody response. Nevertheless, an association between lack of pre-vaccination antibodies and response to vaccination could not be proven in the present study but the small number of dogs lacking pre-vaccination antibodies might limit statistical assessment. The low number of vaccine responders questions the necessity of modified live CAV-2 re-vaccination in adult healthy dogs; however, the association between lack of antibodies (and thus possible lack of protection) and increasing time since the last vaccination (>3 years) suggests that triennial re-vaccinations according to current guidelines should be performed (if vaccination is recommended at all) or antibodies should be measured. A flexible administration of vaccine components would be desirable and CAV-2 should be offered as a single-component vaccine rather than be included in multi-component products.

Further animal-related factors have been described to affect the level of immune response in the past, e.g., size-related differences after CPV vaccination [21] and after rabies vaccination [52]. Although not statistically significant, there was a trend towards a better response to vaccination in dogs with a lower body weight in the present study. The most likely cause is a different deposition of subcutaneous fat which could sequester the vaccine virus. Similar to human medicine, age-related changes in the immune system might influence the response to vaccination in older animals such as described, e.g., for dogs’ response to vaccination against rabies [53] and CPV [54]. The present study is the first study that investigated whether response to CAV vaccination is influenced by the dog’s age but an association could not be demonstrated. Overall, the small number of dogs that responded to vaccination likely limited assessment of related factors.

Mild and transient VAAEs after vaccination occurred in many dogs; owners were very well informed and advised to pay special attention to VAAEs and it is possible that the recorded signs are usually not reported. Mild transient VAAEs are considered to be normal consequences of vaccination and can be caused by the initiation of the immune response and cytokine production as well as by the vaccine virus, adjuvants, or endotoxins [17,55,56,57,58]. Mild lymphadenopathy was more common in dogs with lack of pre-vaccination antibodies indicating that a (low-grade) infection caused by MLV and a more pronounced immune response are more common in these dogs since MLV is not neutralized by pre-existing antibodies. However, none of the systemic VAAEs were associated with an antibody response after vaccination.

## 5. Conclusions

The majority of dogs (87.3%) had antibodies against CAV in the present study, even those that had not received a complete vaccination series. Dogs were more likely to lack antibodies if their last CAV-2 vaccination had been performed >3 years ago. A response to vaccination (≥4-fold titer increase) was observed in only 6.2% of the dogs. Dogs from urban areas were more likely to respond to vaccination than dogs from rural areas. Considering the results of the present study and the fact that dogs lacking antibodies are likely not protected against the disease, adult dogs should receive re-vaccinations with modified live CAV-2 according to the current guidelines every 3 years (if vaccination against CAV is indicated). However, even better, antibody measurement should be performed before vaccination to avoid re-vaccination of dogs that already have antibodies. Further studies are necessary to determine the role of non-responding and a potential breed predisposition of Retrievers to CAV-2 vaccination.

## Figures and Tables

**Table 1:** Factors associated with lack of antibodies against canine adenovirus in univariate and multivariate analysis.

Variable	Factor	Number of Dogs in Each Group	Dogs with Lack of Pre-Vaccination CAV ^1^ Antibodies (%)	Univariate Analysis	Multivariate Analyses		
				p ^2^	*p*	Odds Ratio	95% CI ^3^
Age	<2 years	7/97	1/7 (14%)	0.421	-		
	2 ≤ 9 years	75/97	5/75 (7%)	Ref.^4^			
	>9 years	15/97	1/15 (7%)	1.000			
Sex	Female	57/97	5/57 (9%)	0.696	-		
	Male	40/97	2/40 (5%)				
Weight	<10 kg ^6^	16/97	2/16 (13%)	**0.006**	eliminated ^a^		
	10-20 kg	23/97	0/23 (0%)				
	20–30 kg	31/97	0/31 (0%)				
	>30 kg	27/97	5/27 (19%)				
Neutering status	Intact	47/97	3/47 (6%)	1.000	-		
	Neutered	50/97	4/50 (8%)				
Origin	Breeder	33/97	4/33 (12%)	0.604	-		
	Private	23/97	1/23 (4%)				
	Shelter	13/97	1/13 (8%)				
	Humane society	28/97	1/28 (4%)				
Environment	Urban	56/97	4/56 (7%)	1.000	-		
	Rural	41/97	3/41 (7%)				
Lifestyle	Family	70/97	5/70 (7%)	0.409	-		
	Breeding	9/97	0/9 (0%)				
	Farm	4/97	1/4 (25%)				
	Utility	14/97	1/14 (7%)				
History abroad	Yes	65/97	5/65 (8%)	1.000	-		
	No	32/97	2/32 (6%)				
Housing conditions	Other dogs/cats	62/97	4/62 (6%)	0.700	-		
	No other dogs/cats	35/97	3/35 (9%)				
Daily contact with other dogs	<2	20/97	3/20 (15%)	0.204	-		
	3–5	58/97	4/58 (7%)				
	>5	19/97	0/19 (0%)				
Time since last vaccination	1–3 years	77/97	2/77 (3%)	**0.003**	Ref. ^4^	n.a. ^5^	n.a.
	>3–5 years	15/97	3/15 (20%)		**0.020**	**9.375**	**1.416–62.064**
	>5 years	5/97	2/5 (40%)		**0.006**	**25.000**	**2.571–243.006**
Vaccination status	Complete vaccination series	19/97	1/19 (5%)	1.000	-		
	Incomplete vaccination series	78/97	6/78 (8%)				

^1^ Canine adenovirus; ^2^ *P*-value; ^3^ Confidence interval; ^4^ Reference value; ^5^ not applicable; ^6^ kg. Bold values indicate statistical significance. ^a^ The factor was eliminated by the variable selection process of the logistic regression model and was thus not associated with the lack of pre-vaccination antibodies.

**Table 2:** Factors associated with the response to vaccination (≥4-fold titer increase) after modified live canine adenovirus-2 vaccination in univariate analysis.

Variable	Factor	Number of Dogs in Each Group	Dogs with CAV ^1^ Titer Increase (%)	Univariate Analysis		
				*p* ^2^	Odds Ratio	95% CI ^3^
Age	<2 years	7/97	1/7 (14%)	0.364	-	-
	2≤9 years	75/97	4/75 (5%)	Ref.		
	>9 years	15/97	1/15 (7%)	0.580		
Sex	Female	57/97	3/57 (5%)	0.688	-	-
	Male	40/97	3/40 (8%)			
Weight	<10 kg ^4^	16/97	3/16 (19%)	0.068	-	-
	10-20 kg	23/97	1/23 (4%)			
	20-30 kg	31/97	0/31 (0%)			
	>30 kg	27/97	2/27 (7%)			
Neutering status	Intact	47/97	2/47 (4%)	0.678	-	-
	Neutered	50/97	4/50 (80%)			
Origin	Breeder	33/97	3/33 (9%)	0.839	-	-
	Private	23/97	1/23 (4%)			
	Shelter	13/97	0/13 (0%)			
	Humane society	28/97	2/28 (7%)			
Environment	Urban	56/97	6/56 (11%)	**0.037**	**-** *	**-** *
	Rural	41/97	0/41 (0%)			
Lifestyle	Family	70/97	6/70 (9%)	0.829	-	-
	Breeding	9/97	0/9 (0%)			
	Farm	4/97	0/4 (0%)			
	Utility	14/97	0/14 (0%)			
History abroad	Yes	65/97	4/65 (6%)	1.000	-	-
	No	32/97	2/32 (6%)			
Housing conditions	Other dogs/cats	62/97	4/62 (6%)	1.000	-	-
	No other dogs/cats	35/97	2/35 (5%)			
Daily contact with other dogs	<2	20/97	2/20 (10%)	0.281	-	-
	3-5	58/97	2/58 (3%)			
	>5	19/97	2/19 (11%)			
Time since last vaccination	1-3 years	77/97	5/77 (6%)	1.000	-	-
	>3-5 years	15/97	1/15 (7%)			
	>5 years	5/97	0/5 (0%)			
Vaccination status	Complete vaccination series	19/97	1/19 (5%)	1.000	-	-
	Incomplete vaccination series	78/97	5/78 (6%)			
Pre-vaccination CAV titer	≥10	90/97	5/90 (6%)	0.370		
	<10	7/97	1/7 (14%)			

^1^ Canine adenovirus; ^2^ *P*-value; ^3^ confidence interval; ^4^ kilogram. Bold values indicate statistical significance. * Analysis not possible as one row is filled with zero.

**Table 3:** Factors associated with systemic vaccine-associated adverse events occurring in 34 dogs after modified live canine adenovirus-2 vaccination in univariate analysis.

Variable	Factor	Number of Dogs with All Systemic VAAEs ^1^ in Each Group (%)	Univariate Analysis			Lethargy in Each Group (%)	Univariate Analysis			Peripheral lymphadenopathy in Each Group (%)	Univariate Analysis			Gastro-Intestinal Signs in Each Group (%)	Univariate Analysis		
			*P* ^2^	Odds Ratio	95% CI ^3^		*P* ^2^	Odds Ratio	95% CI		*P*	Odds Ratio	95% CI		*P*	Odds Ratio	95% CI
Age	<2 years	4/7 (57%)	0.216	-	-	3/7 (43%)	0.135	-	-	2/7 (29%)	0.806	-	-	2/7 (29%)	0.349	-	-
	2≤9 years	27/75 (36%)				19/75 (25%)				14/75 (19%)				10/75 (13%)			
	>9 years	3/15 (20%)				1/15 (7%)				3/15 (20%)				3/15 (20%)			
Sex	Female	19/57 (33%)	0.673	-	-	13/57 (23%)	0.813	-	-	12/57 (21%)	0.797	-	-	9/57 (16%)	1.000	-	.
	Male	15/40 (38%)				10/40 (25%)				7/40 (18%)				6/40 (15%)			
Weight	<10 kg	7/16 (44%)	0.782	-	-	6/16 (38%)	0.500	-	-	4/16 (25%)	0.371	-	-	4/16 (25%)	0.394	-	-
	10–20 kg	8/23 (35%)				4/23 (17%)				5/23 (22%)				5/23 (22%)			
	20–30 kg	9/31 (30%)				6/31 (19%)				3/31 (10%)				3/31 (10%)			
	>30 kg	10/27 (37%)				7/27 (26%)				7/27 (26%)				3/27 (11%)			
Neutering status	Intact	15/47 (32%)	0.671	-	-	10/47 (21%)	0.639	-	-	10/47 (21%)	0.800	-	-	7/47 (15%)	1.000	-	-
	Neutered	19/50 (38%)				13/50 (26%)				9/50 (18%)				8/50 (16%)			
Pre-vaccination CAV ^3^ titer	≥10	31/90 (34%)	0.693	-	-	21/90 (23%)	0.668	-	-	15/90 (17%)	**0.026**	**6.480**	**0.988–48.842**	14/90 (16%)	1.000	-	-
	<10	3/7 (43%)				2/7 (29%)				4/7 (57%)				1/7 (14%)			
Response to vaccination (≥4-fold titer-increase)	Yes	2/6 (33%)	1.000	-	-	1/6 (17%)	1.000	-	-	1/6 (17%)	0.370	-	-	2/6 (33%)	0.231	-	-
	No	32/91 (35%)				22/91 (24%)				18/91 (20%)				13/91 (14%)			

^1^ Vaccine-associated adverse events; ^2^ P-value; ^3^ confidence interval; ^4^ kilogram; ^5^ canine adenovirus. Bold values indicate statistical significance.

**Table 4:** Dogs lacking antibodies against canine adenovirus on day 0.

Dog	Signalment	Weight	Origin, Lifestyle	Environment	Daily Contact to Other Dogs	Previous Vaccinations	Time Since Last Vaccination	Complete Vaccination Series *	CAV ^1^ Antibody Titer			VAAEs ^2^ after Vaccination
									Day 0	Day 7	Day 28	
1	Golden Retriever, 8.5 y ^3^, female, intact	41 kg ^4^	Breeder, family dog	Urban	< 2	8, 11, 16 w, 5, 3 y	5 y	No	<10	160	320	Yes: Mild gastrointestinal signs on day 0–7 after vaccination
2	Mix, 13.8 y, male, neutered	38 kg	Private, farm dog	Rural	<2	15 w, 1, 2, 3, 4, 5, 6, 7, 8 y	5 y	No	<10	<10	10	No
3	Labrador Retriever, 1.3 y, female, intact	30 kg	Breeder, family dog	Rural	3-5	14, 17, 20 w	1 y	No	<10	<10	10	No
4	Golden Retriever, 3.3 y, male, intact	33 kg	Breeder, utility dog	Rural	3-5	12, 15 w	3 y	No	<10	<10	<10	Yes: Mild lethargy on day 0–7 after vaccination
5	Mix, 7.4 y, female, neutered	7 kg	Humane society, family dog	Urban	3-5	7, 11 w, 6.2 y	6 y	No	<10	<10	<10	No
6	Labrador Retriever, 8.6 y, female, intact	30 kg	Shelter, family dog	Urban	<2	3, 5, 8.5 y	3 y	No	<10	<10	<10	Yes: Mild lethargy on day 0–7 after vaccination
7	Labradoodle, 6.2 y, female neutered	8 kg	Breeder, family dog	Urban	3-5	12, 15, 17 w, 1, 4 y	2 y	Yes	<10	<10	<10	No

^1^ canine adenovirus; ^2^ vaccine-associated adverse events; ^3^ years;^4^ kilogram; ^5^ weeks. * Vaccination status was considered complete if dogs had received a completed primary vaccination (with a last booster at 16 weeks or older and again 11–13 months later) and regular re-vaccinations at least every 3 years.
